# Association of the maternal 14-bp insertion/deletion polymorphism in the histocompatibility leukocyte antigen G gene with recurrent implantation failure

**Published:** 2014-09

**Authors:** Mahbubeh Enghelabifar, Somaiieh Allafan, Jina Khayatzadeh, Khadijeh Shahrokh Abadi, Mohammad Hasanzadeh Nazarabadi, Fahimeh Moradi, Nozhat Musavifar, Mohsen Jalali, Majid Mojarrad

**Affiliations:** 1 *Department of Biology, Sciences Faculty, Mashhad Branch, Islamic Azad University, Mashhad, Iran.*; 2 *Department of Medical Genetics, Mashhad University of Medical Sciences, Mashhad, Iran.*; 3 *Medical Genetics Research Center, School of Medicine, Mashhad University of Medical Sciences, Mashhad, Iran.*; 4 *Montaserieh Infertility Treatment Center, Mashhad University of Medical Sciences, Mashhad, Iran.*

**Keywords:** *Recurrent implantation failure*, *Assisted reproductive techniques*, *HLA*-*G*

## Abstract

**Background:** Implantation failure of blastocyst is one of the main reasons of failure to become pregnancy following use of Assisted Reproductive Techniques. HLA-G, one of the non-classic HLA subtypes, seems to have a vital role in neutralizing of mother immune system. According to importance of ins/del polymorphism of HLA-G in regulation of HLA-G expression, it seems that this polymorphism has an important effect in immune response against embryo, and so success of embryo implantation.

**Objective:** In this experiment we try to evaluate association of HLA-G ins/del polymorphism with risk of occurrence of RIF in ART treated infertile women.

**Materials and Methods: **To evaluating insertion/deletion polymorphism association with RIF we design a case-control study. We select 40 women with history of recurrent failure to become pregnant following IVF as RIF case group. Forty women with pregnancy following IVF were selected as control. Members of both groups were assessed to rule out of anatomical, immunological and known genetical cause of infertility. Presence of 14 bp insertion/deletion alleles was assessed using PCR-PAGE technique. The data were analyzed by means of SPSS software using Chi-Square tests at the significant level of p<0.05.

**Results: **Our data shows that frequency of heterozygote genotype (ins/del) was significantly higher in case group. Furthermore presence of HLA-G insertion/deletion genotype shows association with increase of implantation failure risk by 3.85 fold.

**Conclusion:** According our results, Heterozygote genotype of ins/del leads to increase of RIF risk. It seems that by genotyping of HLA-G polymorphism, we can predict risk of implantation failure in infertile women after use of ART.

## Introduction

Despite considerable advances in the field of assisted reproductive techniques (ART) success rate of implantation after embryo transfer doesn’t improve as high as acceptable. It is estimated that up to 85% of reproductive assisted techniques derived embryos couldn’t implant into the endometrium after transferring into the uterus (1). By definition, not occurring of pregnancy after at least 2 embryo transfer experiment (using embryos in appropriate morphology and division stage) gives as recurrent implantation failure (RIF) (2, 3). 

Recurrent implantation failure occurs due to a wide range of different physiological factors such as hormonal disorders, reduced acceptance of endometrium, defects in fetal growth, and abnormal changes in the expression of adhesion molecules, immunological factors and genetic abnormalities. Note that the event RIF and recurrent spontaneous abortion (RSA) largely overlap in terms of etiology; it seems that the results of studies on the RSA can also be extended to the RIF. On average it is estimated that about 40% of recurrent spontaneous abortion (RSA) cases are due to immunological factors (4-6). 

Human leukocyte antigens (HLA) play an important role in the physiology of pregnancy and fetal mother immunologic interaction (7, 8). Briefly, HLA antigen family divided into two major categories, Class I and Class II. HLA-I antigens are divided into two groups: classical and non-classical HLAs. Former class consists of the three major groups named HLA-A and HLA-B and HLA-C (9). These antigens express on the surface of all nucleated cells and present cellular antigens to CD^8+^ T lymphocytes which eventually leads to activation of the cellular immunity system against foreign or neoplastic antigens. The latter class including HLA-G, HLA-E and HLA-F antigen family, have a more restricted range of tissue expression (10).

HLA-G, a single locus gene located on chromosome 6p21.3, is one of the most important genes in reproductive physiology. This gene is a non-classical HLA-I family member and has a limited range of tissue expression. High expression levels of this gene in endometrial tissue, suggests the importance of HLA-G in endometrial physiology along pregnancy (11). So far, seven alternative spliced isoforms of HLA-G proteins have been identified, 4 membrane bounded and 3 soluble isoforms (12). HLA-G plays an interesting important role in the inhibition of cellular immunity via inhibition of T lymphocyte and monocyte activation and also apoptosis of CD^+8^ T Lymphocytes. This function of HLA-G has a vital importance in the preservation of implanted blastocyst health in endometrium (8). HLA-G is expressed by oocyte surrounding decidual cells and affects oocyte maturation. In vitro fertilized three-day embryos secreting HLA-G has more chance to be accepted by uterine endometrium (13). 

Several studies show that higher expression of HLA-G by Blastocysts has a significant concordance with a higher success rate of implantation (14). However in clinical context sHLA-G secreted from preimplanted embryo cannot be measured. Unlike classical HLA class I genes, HLA-G has relatively little polymorphism in its sequence. These polymorphisms scattered in coding and non-coding sequences of gene which lead to either protein isoforms or change in expression level of native HLA-G protein. These polymorph alleles have not dominant or negative effects on gene function however these alleles have predisposing effect on some immune response related process such as implantation failure or spontaneous abortion. Insertion or deletion of a 14bp track in 3’ untranslated region of HLA-G mRNA, 14ins/del polymorphism, is shown to have effect on translational activity of HLA-G and eventually leads to change in HLA-G protein expression levels. This change is not classical deleterious mutation but affect susceptibility to some multifactorial diseases. The association between ins/del polymorphism allels of HLA-G and occurrence of RSA have been studied in several population (15). 

majority of these documents suggest a significant association between this polymorphism and increased risk of fetal loss (11, 16). However, A few number of investigate association of ins/del polymorphism and recurrent implantation failure following assisted reproductive techniques such as IVF. *Sipak-Szmigiel et al *demonstrate that including the 14 bp sequence into HLA-G gene is associated with increasing risk of IVF failure (17). Furthermore, In Danish population homozygote 14 bp ins genotype of HLA-G gene is associated with increased risk of implantation failure (18).

In this study the association between this polymorphism and implantation failure in Iranian population was examined.

## Materials and methods

**Patients**

Study was performed as a case-control experiment. The study was approved by the Ethics Committee of Mashhad University of Medical Sciences and all patients gave their written informed consent. Forty infertile women with a history of idiopathic recurrent implantation failure following IVF from Montaserieh Infertility Center, Mashhad, Iran, were included into study. The RIF was defined as at least two failed IVF-embryo transfer, using at least 6 appropriate cleaved embryos. Serum βhCG levels lower than 5mIU/mL two weeks after embryo transfer was defined as implantation failure criterion. 

Semen samples of all male partners were assayed to exclude sperm abnormality. Women were examined by ultrasonography for detection of any anatomical abnormalities of the genitourinary tract and endometriosis. Presence of active viral infections such as cytomegalovirus, Rubella and toxoplasmosis were ruled out using an immunologic test on blood samples. Thyroid hormone levels were evaluated in blood samples to exclude hormonal abnormalities. Presence of immunological risk factors such as anti DNA antibodies, antiphospholipid antibodies were examined. Furthermore couples carrying any karyotypic abnormalities, thrombophilic mutations and also any identified single gene disorder were excluded from study. Forty women with successful implantation following IVF-embryo transfer were included as a control group. 

**Genotyping**

Whole peripheral blood was taken and DNA extraction was performed using spin column based DNA extraction kit (GENET BIO). Quantity and quality of DNA samples were evaluated using spectrophotometry and agarose gel electrophoresis. To discrimination of ins/del alleles PCR reaction was performed using following primers: HLAGf: 5′-GGAA GGAATGCAGTTCAGCATGA-3′ and HLAGr: 5′-TGGATGGGCTGTTTAAAGTGTCAC-3′.

PCR was performed in 25µl reaction containing 1X PCR buffer, 2mM MgCl2, 0.05 mM of each dNTPs, 0.4 μM of each primer, 1 unit of Taq DNA polymerase and 100 ng of template DNA. PCR reaction was performed in 2720 ABI thermal cycler which is programmed for 30 cycles of denaturation at 94ºC for 30s, 63ºC for 40s and 72ºC for 2min; an additional step of denaturation at 95ºC for 10min preceded the first cycle and another step of extension at 72ºC for 7 min was extended at the end of reaction. PCR products were electrophoresed on 2% agarose gel and after ethidium bromide staining PCR product bands were visualized under UV light. Ins/del alleles were discriminated using polyacrylamide gel electrophoresis followed by silver nitrate staining.

**Statistical analysis**

Genotyping results were analyzed to determine differences between group allele and genotype frequencies using “Pearson Chi-Square” test.

P<0.05 was defined as significant frequency difference between the two groups. To evaluate the relationship between either alleles or genotypes with risk of having a recurrent embryo implantation failure, Odd Ratio was calculated for each genotype and allele against others. Association was expressed as odds ratios (OR) or risk estimates with 95% confidence intervals (CI). Association was considered significant at p<0.05. All analyses were performed using SPSS software (Statistical Package for the Social Sciences, version 11.5, SPSS Inc, Chicago, Illinois, USA) 

## Results

**Figure 1:**
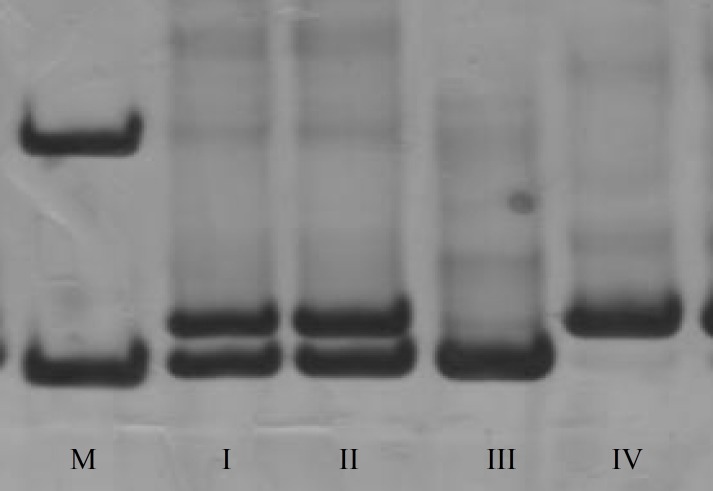
Allele discrimination using 8% polyacrylamide gel electrophoresis. Samples I and II are heterozygote genotype, lane III has an ins/ins homozygote genotype and lane IV shows del/del homozygote genotype. lane M is 100 bp DNA size marker.

The results of the PCR-PAGE are illustrated in figure 1. As shown in the figure 1, the presence of a unique 210 bp band in a lane indicates homozygous del/del genotype while Presence of a 224 bp band is a sign of homozygous ins/ins genotype. Heterozygote samples show both 210bp and 224bp bands in PAGE result. Allelic and genotypic frequencies are given in table I. From 40 RIF suffering patients, 7 patients have homozygote genotype for insertion allele, which is equivalent to 17.5% of the population, while 9 out of 40 controls, 22.5%, have this genotype. 

Ins/del genotype in RIF and control population were 82.5% and 55% respectively. There was no del/del homozygote in RIF group against 27.5% in the control group. Using Pearson Chi-Square test no allelic frequency difference was seen between the two groups. However genotype distribution between two groups was significantly different (Table I). As shown in table I, ins/del genotype are associated with increased risk of implantation failure, odds ratio of 3.85.

**Table I:** Genotype and allele frequency distribution among different groups

	**Healthy control**	**RIF case**	**Odds ratio (95% CI)**	**p-value**
Ins/ins	9 (22.5%)	7 (17.5%)	0.73 (0.36-1.4)	0.377
Ins/del	22(55%)	33(82.5%)	3.85 (2.01-7.38)	<0.0001*
del/del	8 (20%)	0 (0%)	Not computable	Not computable
del allele	0.52	0.41	reffrence	
ins allele	0.48	0.59	1.55 (0.89-2.72)	0.156**

Ins: insertion allele

del: deletion allele

* Pearson Chi-Square.

** Chi-square with Yates correction

## Discussion

to date a little number of studies have been carried out on the association of polymorphisms ins/del HLA-G genotypes and embryo implantation failure following IVF. The correlation between high levels of HLA-G expression in preimplantation embryos and increase of successful embryo implantation chance represents HLA-G as a key factor in determining the fate of preimplantation embryos (19). Analysis of serum and amniotic levels of HLA-G shows that HLA-G has a distinct expression change pattern along pregnancy development (14). As pregnancy progresses HLA-G expression levels in amniotic fluid decreases. So maternal immune system tolerance against fetus decreases and eventually leads to rejection of the fetus, childbirth. Abnormal reduction of HLA-G expression level (either soluble or membrane-bound form) in the early months of pregnancy may lead to an increased risk of immunologic response of the mother immune system against the fetus, resulting in abortion (20).

In case of abnormal low expression levels of HLA-G by either preimplantation embryo or mother, exaggerated immune response leads to the inability of the blastocyst to implanting into the endometrium. Evidence that HLA-G-mediated molecular mechanisms involved in termination of pregnancy have been well reviewed by Cecati* et al* (20). Several studies show that specific types of HLA-G alleles are associated with increased risk of recurrent spontaneous abortion (21). HLA-G 14bp insertion/deletion polymorphism (Ins/del) widely studies in pregnancy physiology area (22). Several theories have been proposed about the mechanisms of the effects of this polymorphism in pregnancy. 

Most justified in this case is the importance of polymorphism in the mRNA structure. According to this theory, despite the location of this polymorphism, in 3’ untranslated region of mRNA, it leads to decrease of mRNA stability and eventually, decrease of HLA-G protein production. This reduction leads to immune system intolerance against embryo and thereby increase the likelihood of embryo implantation failure or abortion of implanted fetal (23). Studies conducted in different populations show different, sometimes contradictory results. Hviid *et al* investigate frequency of HLA-G ins/del alleles in Indian normal and RSA suffered women (18). Results of this study suggest association between HLA-G ins/del genotype and RSA. furthermore, in a survey conducted by the same group on the association of this polymorphisms and successful IVF, IVF failure was associated with the ins/ins homozygous genotype (16).

The study which was conducted in 2004 by Tripathi *et al* suggest heterozygous genotype associated with increased risk of RSA (15). The results of this study were confirmed by several other experiments in various populations (24). however Sipak-Szmigie *et al* in a study on Polish RSA does not found any association between this polymorphism and RSA (25). Vargas *et al *in 2011 demonstrate that the combination of immunological haplotypes and ins/del HLA-G genotype is associated with increased risk of RSA (26).

In present experiment we investigate association between ins/del polymorphism of HLA-G and risk of recurrent implantation failure. The results confirm the relationship between ins/del HLA-G genotype and increased risk of implantation failure. The results obtained in this study is in concordance with the results of Tripathi *et al* and Hviid *et al*, while is in conflict with the results of other studies. This finding can be explained due to similarity of genetic background between Indian and Iranian population.
